# Clinical and Genomic Insights into Antifungal Resistance in *Aspergillus* Isolates from Thailand

**DOI:** 10.3390/microorganisms13112495

**Published:** 2025-10-30

**Authors:** Thanyarat Onchan, Nattapong Langsiri, Arsa Thammahong

**Affiliations:** 1 Center of Excellence in Antimicrobial Resistance and Stewardship, Department of Microbiology, Faculty of Medicine, Chulalongkorn University, Bangkok 10330, Thailand; nawthanyarat@gmail.com; 2 Department of Transfusion Medicine and Clinical Microbiology, Faculty of Allied Health Science, Chulalongkorn University, Bangkok 10330, Thailand; nutleng1150@gmail.com; 3 Center of Excellence of Medical Mycology Diagnosis, Chulalongkorn University, Bangkok 10330, Thailand

**Keywords:** *Aspergillus*, antifungal resistance, Thailand

## Abstract

*Aspergillus* species are major opportunistic pathogens responsible for invasive aspergillosis, with antifungal resistance posing increasing challenges to their treatment worldwide. We investigated the antifungal susceptibility and genomic features of *Aspergillus* isolates from sterile clinical specimens collected at a tertiary hospital in Thailand between January and December 2023. In total, 24 isolates were identified via culture and tested for amphotericin B and voriconazole susceptibility using CLSI M38 broth microdilution, and whole-genome sequencing was performed on selected isolates to characterize resistance mechanisms. *Aspergillus fumigatus* was the most frequent species (54%), followed by *A. flavus* (29%) and other less common species. Voriconazole exhibited potent activity against most isolates, although two *A. fumigatus* strains showed elevated MICs (2–4 µg/mL), meeting resistance thresholds. One isolate (CUAFU23) was confirmed to harbor the *cyp51A* TR34/L98H mutation, marking the first identification of this canonical azole resistance mechanism in a clinical specimen from Thailand and supporting earlier environmental findings of azole-resistant *A. fumigatus* carrying the same allele. Genomic profiling of CUAFU23 further revealed subtle but distinct shifts in domain composition compared with susceptible strains, suggesting broader adaptive changes. The above findings underscore the emergence of azole-resistant *A. fumigatus* in Thailand and highlight the importance of ongoing surveillance using combined phenotypic and molecular approaches.

## 1. Introduction

*Aspergillus* species are ubiquitous environmental molds that cause a wide range of opportunistic infections in humans, collectively referred to as aspergillosis. Among them, *Aspergillus fumigatus* is the most common invasive pathogen, but other species, such as *A. flavus*, *A. terreus*, *A. niger*, and *A. nidulans*, are also significant etiologic agents, particularly in specific geographic regions and patient populations [1,2]. *Aspergillus* species can cause invasive aspergillosis [2], chronic pulmonary aspergillosis (CPA), and allergic bronchopulmonary aspergillosis (ABPA), with IA being the most severe form, associated with mortality rates of 30–60% despite antifungal treatment [3]. The World Health Organization has classified *A. fumigatus* as a critical priority fungal pathogen, underscoring the urgency of addressing antifungal resistance (WHO fungal priority pathogens list to guide research, development, and public health action. Geneva: World Health Organization; 2022).

Azole antifungals, including itraconazole, voriconazole, and posaconazole, target the ergosterol biosynthesis enzyme CYP51A and are the first-line drugs for treating and preventing aspergillosis [4]. However, azole resistance has emerged as a pressing clinical concern worldwide, linked to both medical and environmental exposures [1,2]. Resistance is commonly mediated by tandem repeats in the promoter region of *cyp51A* (e.g., TR34/L98H, TR46/Y121F/T289A), which promote gene overexpression, combined with amino acid substitutions that reduce azole binding affinity [1,2]. Additional mutations have been identified in *cyp51B* and *hmg1*, regulators of sterol biosynthesis, further expanding the resistance repertoire [1,2,5].

Notably, increasing numbers of azole-resistant *A. fumigatus* (AR*Af*) isolates lack *cyp51A* mutations entirely, particularly in Japan, suggesting alternative mechanisms of resistance [6], which may include enhanced expression of efflux transporters (e.g., *cdr1B*), abnormalities in mitochondrial function, and altered post-translational modifications [2,3]. Environmental and clinical surveys have revealed substantial geographic heterogeneity in the prevalence and mechanisms of azole resistance; for instance, TR-type mutants are more prevalent in Europe, while non-TR AR*Af* strains are increasingly reported in Asia [6].

Genomic studies have illuminated the diversity of *A. fumigatus* populations worldwide. In Japan, population genomics of 171 strains revealed assignment into six distinct genetic clusters, highlighting a complex evolutionary background for resistance development [6]. Importantly, approximately 43–65% of AR*Af* strains in some regions do not carry *cyp51A* mutations, underscoring the need to characterize alternative resistance pathways [6].

Furthermore, recent evidence suggests that certain azole-resistant *A. fumigatus* populations evolve more rapidly, due to increased mutation rates driven by defects in DNA mismatch repair (MMR) genes [3]. A specific point mutation, *msh6*-G233A, found almost exclusively in strains harboring the TR34/L98H allele, was shown to increase mutation rates up to fivefold without compromising fitness, enhancing the risk of developing resistance to newer antifungal medications, such as olorofim [3]. This mutator phenotype, tightly linked with resistant genotypes, poses a serious threat, as it may accelerate adaptation under antifungal pressure in both clinical and environmental contexts [3]. In summary, the emergence of genetically diverse, environmentally resilient, and mutationally dynamic AR*Af* strains presents a major challenge to antifungal therapy. Continued surveillance, genomic characterization, and mechanistic studies are essential to inform treatment strategies and limit the global spread of antifungal resistance. In this study, our objective is to observe antifungal resistance and mutations in *Aspergillus* species at a tertiary hospital in Thailand.

## 2. Materials and Methods

### 2.1. Fungal Strains, Media, and Conditions

*Aspergillus flavus* ATCC 204304 and *Aspergillus* isolates were cultured on Sabouraud Dextrose Agar (SDA, Oxoid, Thermo Fisher Scientific, Waltham, MA, USA) plates at 37 °C for 3–5 days until adequate conidiation was achieved. The colonies were harvested into distilled water using cell scrapers. The distilled water containing *Aspergillus* spores was filtered through miracloth, and the spores were counted using a hemocytometer.

*Aspergillus* clinical isolates, from sterile sites with proven invasive aspergillosis diagnoses, were obtained from the Mycology Laboratory, Department of Microbiology, Faculty of Medicine, Chulalongkorn University, and King Chulalongkorn Memorial Hospital, from 1 January 2023 to 31 December 2023. Patient characteristics were collected from medical records/charts. Patients with invasive aspergillosis (IA) were classified as having proven, probable, or possible invasive aspergillosis, according to EORTC/MSG criteria [7].

### 2.2. Broth Microdilution Assay

The CLSI broth microdilution M38 method was performed to observe the minimum inhibitory concentrations (MICs) of amphotericin B and voriconazole in *A. flavus* ATCC 204304 and clinical isolates [8]. The assay was performed with three biological replicates.

### 2.3. Sequencing Analysis

Five *Aspergillus* spp. isolates were subjected to whole-genome sequencing and annotation. Raw sequencing data in FAST5 format were base-called using Guppy v6.5.7-1 (Oxford Nanopore Technologies, Oxford, UK), during which both adapter and barcode sequences were trimmed, and samples were demultiplexed. Quality filtering of base-called reads was performed using Filtlong v0.3.0 (https://github.com/rrwick/Filtlong, accessed on 7 June 2025), retaining only reads with a minimum q-score of 15. The identification of *Aspergillus* species was conducted using the EPI2ME wf-metagenomics pipeline (Oxford Nanopore Technologies, v.2.10.1). High-quality reads were used for de novo genome assembly using Flye v.2.9.5 [9], and the resulting assemblies were polished using Medaka (Oxford Nanopore Technologies, Oxford, UK, v.1.7.3), employing corresponding reference genomes from the NCBI RefSeq database [10] for each *Aspergillus* species to guide error correction. The reference genomes used for polishing were *A. fumigatus* strain Af293 (GCF_000002655.1, downloaded on 7 June 2025) and *A. flavus* strain NRRL 3357 (GCF_009017415.1, downloaded on 7 June 2025). Repetitive genomic elements were identified and masked prior to annotation using RepeatMasker v4.1.5 [11]. Gene prediction and functional annotation were performed using Funannotate v.1.8.17 [12]. Ab initio gene prediction was conducted using three independent algorithms: Augustus v.3.5.0 [13], SNAP [14], and GlimmerHMM v.3.0.4 [15]. Initial training for these predictors utilized the pre-trained Ascomycota gene models from the BUSCO (Benchmarking Universal Single-Copy Orthologs) pipeline [16], and final training of Augustus employed the *Aspergillus fumigatus* species model for improved prediction accuracy. Protein evidence was incorporated through homology-based alignment using Diamond v2.1.10 and Exonerate v.2.4.0 [17] against curated fungal protein databases. The results from ab initio predictions and protein alignments were then integrated using EvidenceModeler (EVM) v.2.1.0 [18] to generate high-confidence consensus gene models. Transfer RNA genes were predicted using tRNAscan-SE v.2.0.12 and added to the final gene annotation set. Functional annotation of predicted genes was performed using a combination of curated databases and tools. Protein function was assigned, based on homology, to UniProt, EggNOG, MEROPS (for peptidases), or CAZy (for carbohydrate-active enzymes). The 100 most abundant domains from the PFAM, MEROPS, and CAZyme families were selected to perform hierarchical clustering using scipy.cluster.hierarchy from scipy v1.16.2 modules. The row linkage (between protein domains) was determined using Euclidean distance, and column linkage (between strains) was determined using correlation distance.

### 2.4. Profiling the cyp51A Gene for Phylogenetic Analysis

The *cyp51A* sequence of each isolate was extracted using the Funannotate v.1.8.17 [12] pipeline. The promoter region, including the gene region of *cyp51A*, was extracted using the in-house developed pipeline. The representative sequences of the *cyp51A* gene were selected from the azole-resistant strain *A. fumigatus* (GenBank accession number OQ164735.1, AR23I strain) and the normal MIC strain (GenBank accession number AF338659.1, ATCC 36607 strain) using the GenBank database. The inspections of TR34 in the promoter and the mutation in the *cyp51A* gene were conducted using multiple alignment in MEGA X [19] with the representative sequence of *cyp51A*. The phylogenetic analysis of each region (*cyp51A*) of all studied isolates was conducted with MEGA XII [19] using the Maximum-Likelihood model with a bootstrap value of 1000.

### 2.5. Statistical Analysis

All graphical charts were generated using Prism 8 software (GraphPad Software, Inc., San Diego, CA, USA). Given the limited number of isolates, Pearson’s distance was applied to evaluate inter-strain variation using SciPy version 1.16.2 (Python Software Foundation).

### 2.6. Ethics Statement

This study was approved by the Institutional Review Board (IRB No. 601/67, COE No. 047/2024, approved on 30 July 2024), Faculty of Medicine, Chulalongkorn University, Bangkok, Thailand.

## 3. Results

### 3.1. Aspergillus fumigatus Is the Most Common Isolate from Sterile Clinical Specimens

**Figure 1:**
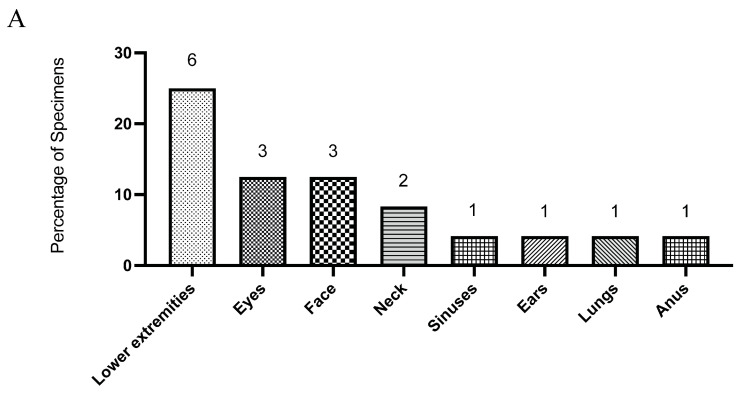

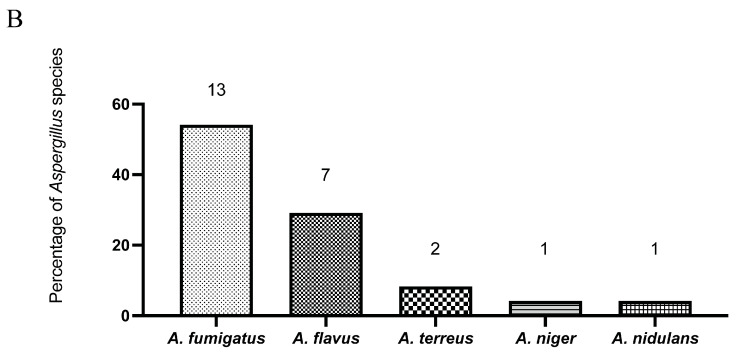
The distribution of clinical specimens is shown by anatomical site (**A**), and the distribution of *Aspergillus* species isolated from these specimens is shown as percentages of all isolates (**B**). Numbers on the graphs indicate the total count of specimens and isolates in each category.

During the study period, 24 *Aspergillus* isolates were recovered from sterile clinical specimens (Figure 1A) and subjected to antifungal susceptibility testing (Table 1). *Aspergillus fumigatus* was the most frequently identified species (*n* = 13; 54.2%), followed by *A. flavus* (*n* = 7; 29.2%). Less common species included *A. terreus* (*n* = 2; 8.3%), *A. niger* (*n* = 1; 4.2%), and *A. nidulans* (*n* = 1; 4.2%) (Figure 1B). Tissue specimens from the lower extremities constituted the most common isolation sites, accounting for approximately one-quarter of all cases, with the remaining isolates distributed across other sterile sites (Figure 1A).

### 3.2. The Minimum Inhibitory Concentrations (MICs) of Voriconazole Remain Low for Most Aspergillus Isolates

Among the 13 *A. fumigatus* isolates, amphotericin B MICs ranged from 1 to 8 µg/mL. A substantial proportion of isolates clustered toward the higher end of this range, with several exceeding the epidemiological cutoff value (ECV). Voriconazole MICs for *A. fumigatus* were generally low, with most isolates inhibited at concentrations between 0.0625 and 0.5 µg/mL. Notably, two isolates exhibited elevated voriconazole MICs of 2 and 4 µg/mL, respectively (Table 1).

For *A. flavus*, amphotericin B MICs ranged between 2 and 8 µg/mL, with the majority of isolates showing values at or above the ECV. In contrast, voriconazole MICs for this species remained uniformly low, ranging from 0.0625 to 0.25 µg/mL, without any evidence of elevated values (Table 1). The two *A. terreus* isolates displayed amphotericin B MICs of 2 and 8 µg/mL, respectively. Voriconazole activity against *A. terreus* was consistent, with both isolates showing MICs of 0.125 µg/mL (Table 1). The single *A. niger* isolate demonstrated reduced susceptibility to amphotericin B, with an MIC of 16 µg/mL. Nevertheless, voriconazole activity was preserved, with an MIC of 0.25 µg/mL (Table 1). The *A. nidulans* isolate exhibited an amphotericin B MIC of 4 µg/mL, while voriconazole was active at 0.125 µg/mL (Table 1).

Two *Aspergillus fumigatus* clinical isolates were recovered from a patient with severe corneal infections who had no prior exposure to antifungal therapy; both isolates exhibited elevated voriconazole MICs (2–4 µg/mL), meeting the CLSI criteria for resistance. Consequently, one of the strains (CUAFU23) was subjected to nanopore sequencing for *cyp51A* mutation analysis compared to three other *A. fumigatus* isolates (CUAFU06, CUAFU15, and CUAFU17) as well as an *A. flavus* isolate (CUAFU09).

### 3.3. The cyp51A Gene of Strain CUAFU23 Carried the TR34/L98H Mutation

**Figure 2:**
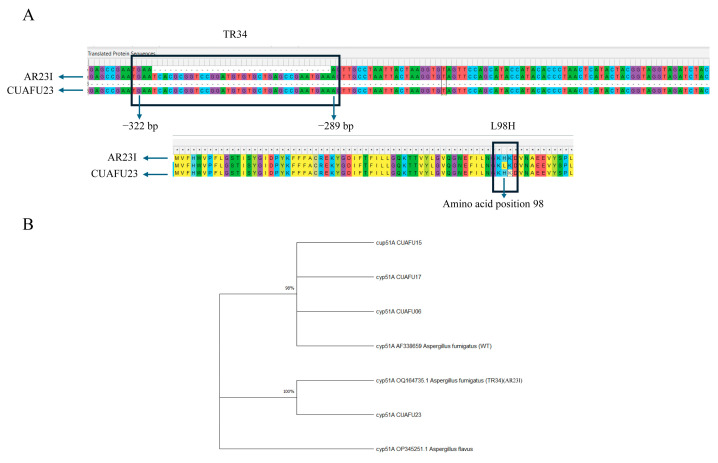
Sequence alignment confirmed the presence of the 34 bp tandem repeat (positions −322 to −289) in the promoter region and the L98H amino acid substitution in the *cyp51A* gene of isolate CUAFU23 compared with the wild-type reference strain (**A**). Phylogenetic analysis further demonstrated that CUAFU23 clustered with strain AR23I, which has previously been reported to carry the same mutation. The *cyp51A* gene of *Aspergillus* was included as an outgroup (**B**).

The *cyp51A* gene of *Aspergillus fumigatus* strain CUAFU23, isolated from a patient with corneal infection, was found to harbor the well-characterized TR34/L98H mutation. Sequence alignment with the wild-type reference strain confirmed the presence of both the 34 bp tandem repeat in the promoter region and the L98H amino acid substitution, demonstrating clear nucleotide and protein-level alterations at the corresponding positions (Figure 2A). Phylogenetic analysis further revealed that CUAFU23 clustered closely with strain AR23I, a previously reported azole-resistant isolate carrying the same mutation (Figure 2B). The above underscore the genetic relatedness of CUAFU23 to known resistant strains and highlight the clinical importance of the TR34/L98H mechanism in mediating azole resistance.

### 3.4. The cyp51A-Mutated Strain Exhibited Distinct Genomic Domain Profiles

To investigate potential genome-wide impacts associated with this mutation, we performed a clustering analysis based on the 100 most abundant protein domains across three major domain families: PFAM, MEROPS, and CAZy. The clustering results revealed a clear distinction between the genomes of *A. flavus* and *A. fumigatus*, with a Pearson’s correlation distance of 0.05, indicating significant divergence in domain composition between the two species.

**Figure 3:**
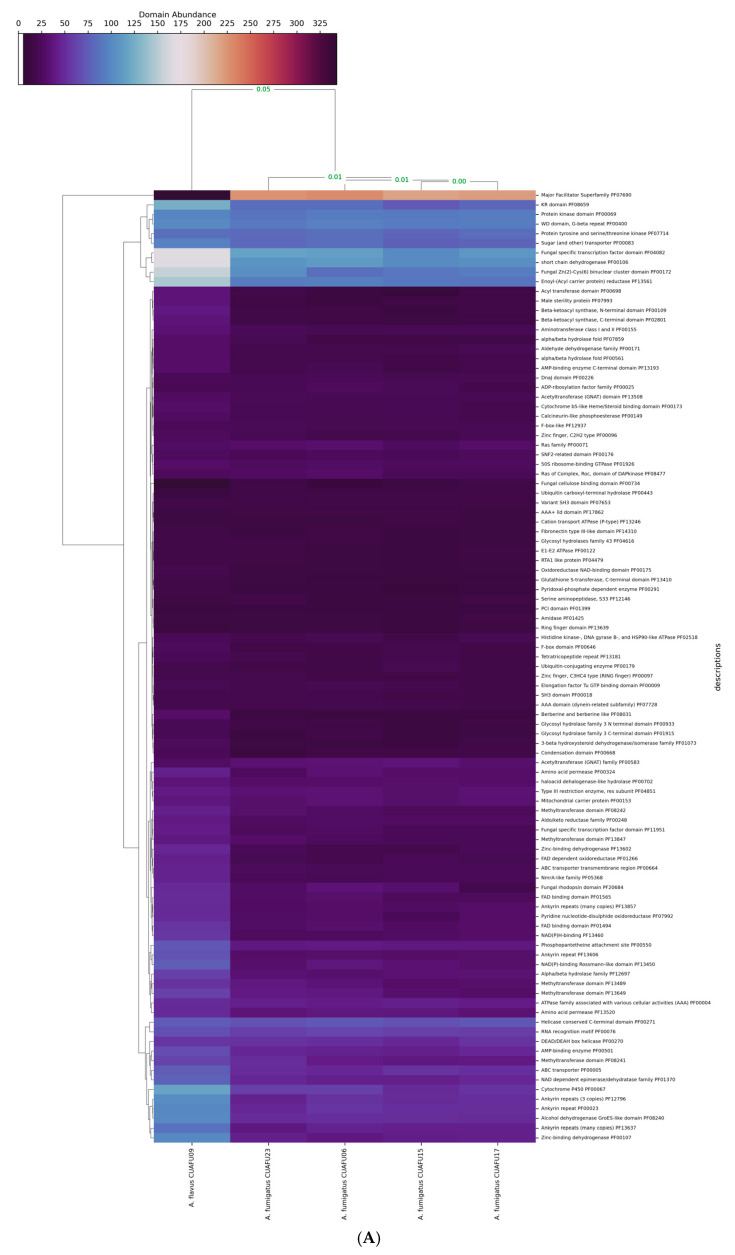

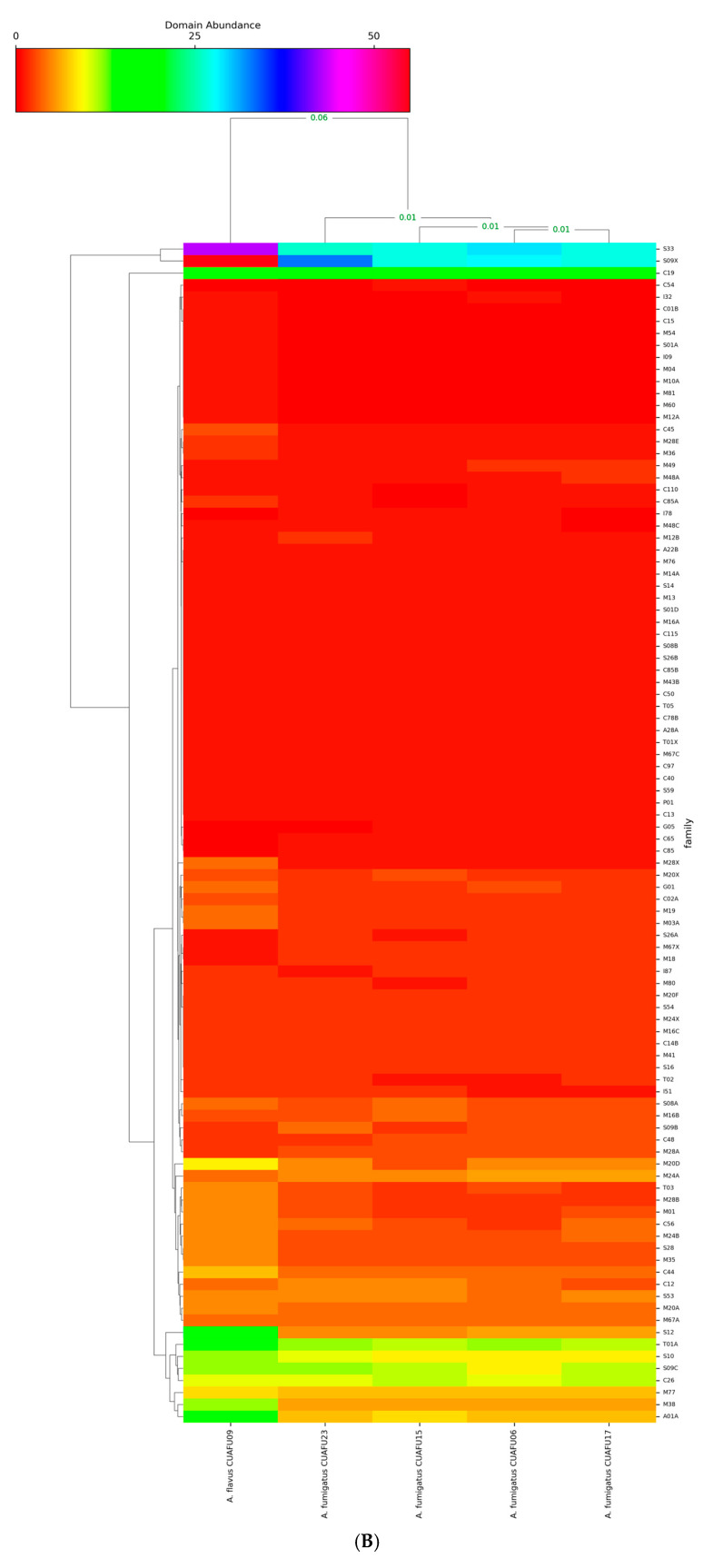

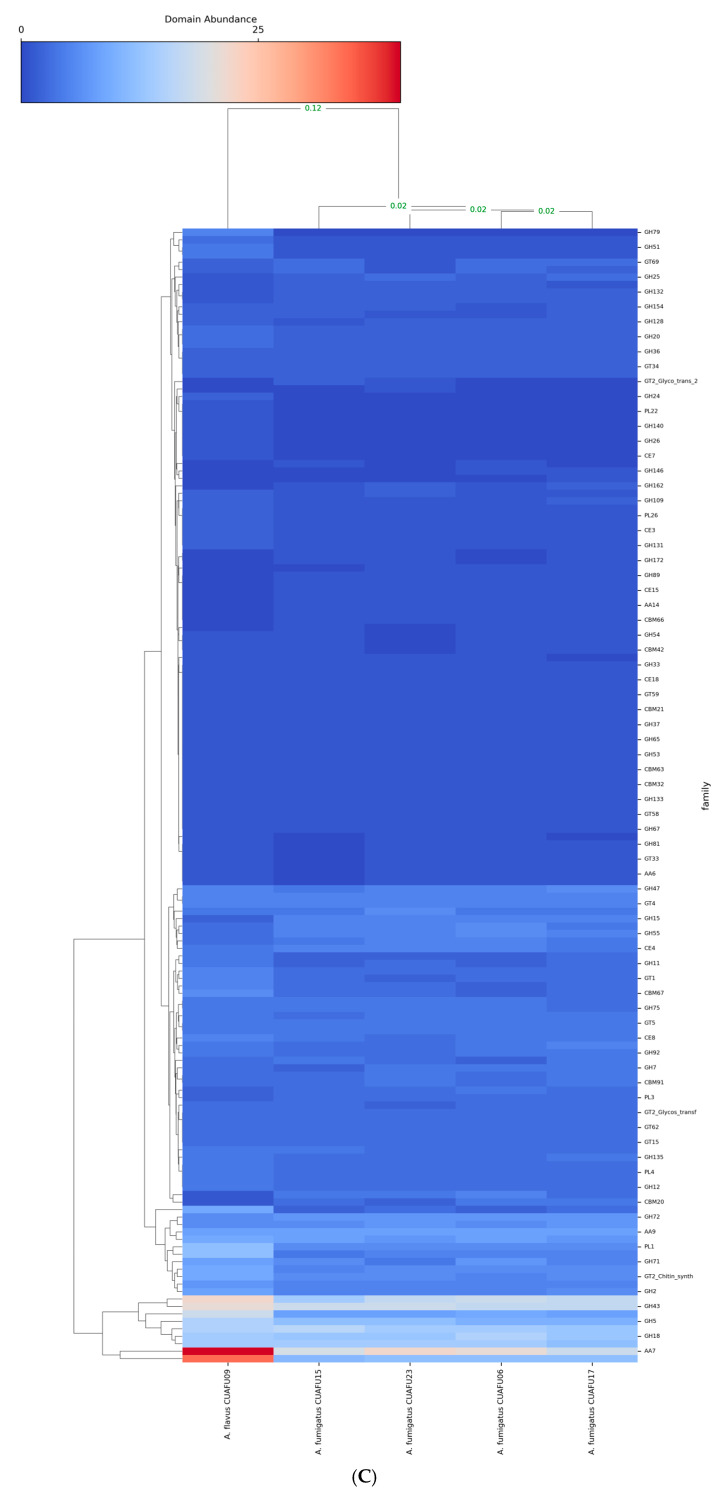
Variation in functional domains were observed in *A. fumigatus* CUAFU23 compared with reference strains. The clinical strain of *A. flavus* was included as an outgroup in the analysis. (**A**) shows differences in PFAM domains, including the Protein kinase domain (PF00069), Amino acid permease (PF00324), Condensation domain (PF00668), Variant SH3 domain (PF07653), Serine aminopeptidase S33 (PF12146), and Fibronectin type III-like domain (PF14310). (**B**) illustrates variation in MEROPS protease domains, where seven domain families (C12, M12B, C48, C19, G05, I87, and S09B) displayed distinct patterns in CUAFU23, suggesting alterations in protein degradation, cellular remodeling, and virulence. (**C**) shows changes in CAZyme domains, where seven glycoside hydrolase and glycosyltransferase families (GH76, GH71, GT20, GT2, GT90, CE1, and GH92) were differentially represented, indicating potential shifts in cell wall remodeling and carbohydrate metabolism.

More subtly, within *A. fumigatus*, the CUAFU23 strain exhibited a slight difference in protein domain abundance compared to other non-mutated strains. Specifically, Pearson’s correlation distance between CUAFU23 and the other *A. fumigatus* strains was measured, and some domains or families have a distance of more than 0.01, found across all domain families (Figure 3). A closer inspection of individual domain contributions highlighted six PFAM domains with notably different counts in CUAFU23 compared to the other strains (correlation distance > 0.01), including the Protein kinase domain (PF00069), Amino acid permease (PF00324), Condensation domain (PF00668), Variant SH3 domain (PF07653), Serine aminopeptidase S33 (PF12146), and Fibronectin type III-like domain (PF14310) (Figure 3A). Similarly, variations in MEROPS protease domains were observed. Seven domain families—C12, M12B, C48, C19, G05, I87, and S09B—showed distinct patterns of different numbers of these families in CUAFU23 (Figure 3B). The above proteases are involved in protein degradation, cellular remodeling, and virulence, suggesting that changes in proteolytic activity may accompany or result from azole resistance. In the case of CAZyme domains, seven glycoside hydrolase and glycosyltransferase families were differentially represented in CUAFU23: GH76, GH71, GT20, GT2, GT90, CE1, and GH92 (Figure 3C).

## 4. Discussion

In this study, using the CLSI broth microdilution method, we characterized the antifungal susceptibility profiles of 24 *Aspergillus* isolates from sterile clinical specimens and further investigated the molecular mechanisms underlying azole resistance in selected strains. *A. fumigatus* was the most prevalent species (54%), consistent with previous reports identifying it as the leading cause of invasive aspergillosis globally [1,20]; however, non-*fumigatus* species, including *A. flavus*, *A. terreus*, *A. niger*, and *A. nidulans*, accounted for 46% of isolates, emphasizing the importance of species-level identification, particularly in endemic regions such as Thailand, where *A. flavus* is frequently encountered [21,22].

Susceptibility testing demonstrated heterogeneous responses to amphotericin B and voriconazole across *Aspergillus* species. Two *A. fumigatus* isolates exhibited elevated voriconazole MICs (≥2 µg/mL), consistent with resistance as defined by CLSI breakpoints [8]. One such isolate (CUAFU23), recovered from a corneal specimen, had a voriconazole MIC of 2 µg/mL and was confirmed to harbor the TR34/L98H mutation in the *cyp51A* gene. This mutation represents a well-established azole resistance mechanism linked to environmental fungicide exposure and associated with clinical treatment failure [4,6].

In Thailand, reports of clinically isolated azole-resistant *A. fumigatus* (AR*Af*) remain scarce. In 2017, Tangwattanachuleeporn et al. screened 308 environmental soils in Thailand and found AR*Af* in 3.25% (10/308), largely due to *cyp51A* TR34/L98H (8/10; 80%), with the remainder due to G54R (2/10; 20%); all showed high itraconazole MICs, with occasional reduced susceptibility to posaconazole and rarely to voriconazole [23]. In 2022, Daloh et al. sampled indoor environments at Walailak University, Thailand (300 swabs), recovering 62 *A. fumigatus* isolates, 17/62 (27.4%) of which were azole-resistant. Among AR*Af*, 82% (14/17) carried hot-spot substitutions around G54, while 18% (3/17) harbored promoter tandem repeats (TR46/TR53) [24]. To our knowledge, our study provides the first evidence of a clinical *A. fumigatus* isolate with TR34/L98H from a corneal specimen in Thailand.

The above finding aligns with reports from Europe and Asia, where TR34/L98H is a dominant mutation in azole-resistant *A. fumigatus* (AR*Af*), particularly in regions with intensive azole fungicide use in agriculture [6,25,26,27,28]; however, the detection of only one TR-type mutant in our cohort may indicate a lower environmental pressure or different resistance dynamics in our local setting. It is worth noting that recent studies in Japan and China have documented increasing numbers of AR*Af* strains without *cyp51A* mutations, implicating alternative mechanisms such as hmg1 mutations, efflux pump overexpression, and mitochondrial alterations [5,6,27].

Interestingly, our genome-wide analysis of protein domain abundance showed subtle, yet consistent, differences in the CUAFU23 strain compared to other azole-susceptible *A. fumigatus* isolates. Specifically, differential abundance of PFAM domains, such as protein kinases and SH3 domains, and variation in the MEROPS and CAZy families, may suggest compensatory changes associated with azole resistance or mutator phenotypes [29,30]. Although these differences may be related to the ability of fungi to adapt to antifungal pressure, virulence modulation, or metabolic remodeling, as suggested in prior multi-omics studies of resistant *A. fumigatus* strains [29], it should be noted that this is still only a hypothesis, and a larger cohort should be further studied for a precise conclusion.

The presence of a TR34/L98H mutation in CUAFU23, along with subtle shifts in domain architecture, is consistent with the hypothesis that this genotype may confer a mutator phenotype. Bottery et al. recently described the msh6-G233A mutation, co-occurring with TR34/L98H, which increases mutation rates without reducing fitness, thus accelerating resistance evolution [3]. Although we did not identify msh6 variants in our strain, the observed genomic distinctions warrant further investigation into the mutational dynamics of resistant isolates in Southeast Asia.

This study has several limitations. First, it was conducted at a single tertiary center with a small number of isolates (*n* = 24) collected over one year, limiting generalizability and precluding temporal trend assessment. Second, antifungal susceptibility testing was restricted to amphotericin B and voriconazole, without evaluation of other triazoles (e.g., posaconazole, isavuconazole), which reduces comparability with regional and international datasets. Third, genotype–phenotype analyses were performed on only a subset of isolates, so additional resistance mechanisms (e.g., *hmg1*, *cyp51B* mutations, efflux pump upregulation) may have been missed. Finally, we did not perform longitudinal environmental surveillance or collect patient-level outcome data, preventing direct linkage between environmental and clinical isolates and limiting inference on clinical impact.

Despite these constraints, we provide the first molecular evidence of a clinical *A. fumigatus* isolate in Thailand harboring *cyp51A* TR34/L98H, underscoring the need for integrated environmental–clinical surveillance and expanded antifungal testing to define the local epidemiology of azole resistance.

## 5. Conclusions

In conclusion, we demonstrate that azole resistance in *A. fumigatus* exists in our clinical setting, with TR34/L98H being one of the resistance mechanisms. Genomic profiling revealed distinct features in resistant isolates, suggesting broader adaptive changes beyond *cyp51A*. These results underscore the importance of integrating antifungal susceptibility testing, molecular diagnostics, and genomic analysis to guide effective treatment strategies and mitigate the spread of resistance.

## Figures and Tables

**Table 1:** Clinical *Aspergillus* isolates were classified as wild-type (WT) or non-wild-type (non-WT) strains, according to the epidemiological cutoff values (ECVs) defined in CLSI M57-S (2022).

Fungal Name (Total Number)/Antifungal	MIC (µg/mL)	MIC Range (µg/mL)	ECV (µg/mL) (CLSI M57S, 2022)	Breakpoint (µg/mL)	WT (%)(N)	Non-W (%)(N)
*Aspergillus fumigatus* (13)						
Amphotericin B	3.15	1–8	2	-	23.08% (3)	76.92% (10)
Voriconazole	0.69	0.0625–4		0.5;1;2 (2024)	85% (11)	15% (2)
*Aspergillus flavus* (7)						
Amphotericin B	4.86	2–8	4	-	14.29% (1)	85.71% (6)
Voriconazole	0.19	0.0625–0.25	2	-	100% (7)	0% (0)
*Aspergillus terreus* (2)						
Amphotericin B	5	2–8	4	-	50% (1)	50% (1)
Voriconazole	0.125	0.125	2	-	100% (2)	0% (0)
*Aspergillus niger* (1)						
Amphotericin B	16	16	2	-	0% (0)	100% (1)
Voriconazole	0.25	0.25	2	-	100% (1)	0% (0)
*Aspergillus nidulans* (1)						
Amphotericin B	4	4	-	-	-	-
Voriconazole	0.125	0.125	-	-	-	-

## Data Availability

The raw data supporting the conclusions of this article will be made available by the authors on request. The sequencing data from this research were submitted to NCBI under the BioProject accession PRJNA1289420.
